# The benthic foraminiferal δ^34^S records flux and timing of paleo methane emissions

**DOI:** 10.1038/s41598-020-58353-4

**Published:** 2020-01-28

**Authors:** C. Borrelli, R. I. Gabitov, M.-C. Liu, A. T. Hertwig, G. Panieri

**Affiliations:** 1 Department of Earth and Environmental Sciences, University of Rochester, Rochester, NY USA; 2 CAGE - Centre for Arctic Gas Hydrate, Environment and Climate, Department of Geosciences, UiT The Arctic University of Norway, Tromsø, Norway; 3 Department of Geosciences, Mississippi State University, Mississippi State, MS USA; 4 Department of Earth, Planetary, and Space Sciences, University of California Los Angeles, Los Angeles, CA USA

**Keywords:** Carbon cycle, Ocean sciences

## Abstract

In modern environments, pore water geochemistry and modelling simulations allow the study of methane (CH_4_) sources and sinks at any geographic location. However, reconstructing CH_4_ dynamics in geological records is challenging. Here, we show that the benthic foraminiferal δ^34^S can be used to reconstruct the flux (i.e., diffusive vs. advective) and timing of CH_4_ emissions in fossil records. We measured the δ^34^S of *Cassidulina neoteretis* specimens from selected samples collected at Vestnesa Ridge, a methane cold seep site in the Arctic Ocean. Our results show lower benthic foraminiferal δ^34^S values (∼20‰) in the sample characterized by seawater conditions, whereas higher values (∼25–27‰) were measured in deeper samples as a consequence of the presence of past sulphate-methane transition zones. The correlation between δ^34^S and the bulk benthic foraminiferal δ^13^C supports this interpretation, whereas the foraminiferal δ^18^O-δ^34^S correlation indicates CH_4_ advection at the studied site during the Early Holocene and the Younger-Dryas – post-Bølling. This study highlights the potential of the benthic foraminiferal δ^34^S as a novel tool to reconstruct the flux of CH_4_ emissions in geological records and to indirectly date fossil seeps.

## Introduction

In marine sediments, sulphate reduction is a fundamental pathway for organic matter remineralization when oxygen is not available and sulphate is abundant (Jørgensen^1^). Because sulphate reduction is also a function of the availability of organic matter, this process is of particular importance in continental margin environments (Jørgensen^1^; D’Hont *et al*.^2^). When coupled with anaerobic oxidation of methane (AOM), sulphate reduction contributes to the degradation of methane (CH_4_) diffusing or advecting from the methanogenic zone or the gas hydrate reservoir (Hoehler *et al*.^3^; Boetius *et al*.^4^). Also, the increase in alkalinity consequent to AOM triggers precipitation of authigenic carbonates, which represent a sink for CH_4_-derived carbon (Aloisi *et al*.^5^). In sediments, the horizon where sulphate and CH_4_ concentrations approach zero is called the sulphate-methane transition zone (SMTZ), and can be located close to the sediment/water interface or tens of meters below the seafloor depending on the depth of the methanogenic zone, transport velocity and consumption rate of CH_4_ and sulphate, and burial rate of organic matter (Knittel and Boetius^6^).

Marine sediments are an important CH_4_ reservoir, in particular because of the presence of gas hydrates, solid ice-like structures of water and gas (Ruppel^7^). The stability of gas hydrates is under intense investigation because of the possible effects of climate change on gas hydrate dissociation (Crémière *et al*.^8^; Hong *et al*.^9^), but a recent study concluded that there was no compelling evidence that CH_4_ derived from gas hydrate dissociation was currently reaching the atmosphere (Ruppel and Kessler^10^).

The benthic foraminiferal carbon isotopic composition (δ^13^C) is one of the tools used to reconstruct paleo CH_4_ emissions (Panieri *et al*.^11^; Consolaro *et al*.^12^; Schneider *et al*.^13^). This approach is based on the ability of foraminifera to register the δ^13^C of CH_4_-related food sources (Rathburn *et al*.^14^; Bernhard and Panieri^15^) or the δ^13^C of dissolved inorganic carbon (DIC), which in sediments characterized by CH_4_ seepage is more negative compared to that of normal seawater (<−48‰ vs. −1−1‰; Torres *et al*.^16^; Ravelo and Hillaire-Marcel^17^) (Panieri *et al*.^11^; Sen Gupta and Aharon^18^; Martin *et al*.^19^). However, some studies do not agree with the possibility to use the benthic foraminiferal δ^13^C as a proxy for paleo CH_4_ seepage (Torres *et al*.^16^; Herguera *et al*.^20^). For example, it was proposed that foraminifera inhabiting CH_4_ seeps might calcify only when CH_4_ seepage is low or absent or during intervals of bottom water intrusion into the sediments (Torres *et al*.^16^). Alternatively, foraminifera might precipitate their shells close to the sediment/water interface, recording primarily the δ^13^C of seawater DIC, and then migrate towards more CH_4_-rich habitats (Herguera *et al*.^20^). In fossil records, very low benthic foraminiferal δ^13^C values can be due to authigenic carbonates that precipitated on fossil shells within the SMTZ at sites characterized by CH_4_ seepage (Consolaro *et al*.^12^; Schneider *et al*.^13^; Torres *et al*.^16^; Panieri *et al*.^21^; Panieri *et al*.^22^; Cook *et al*.^23^). Diagenesis can be a serious issue for the interpretation of geological records, even if recent studies demonstrated the utility of the δ^13^C signature of diagenetically altered foraminifera to reconstruct past migrations of the SMTZ (Schneider *et al*.^13^; Panieri *et al*.^22^). Although the benthic foraminiferal δ^13^C proxy remains controversial, it is still the main approach used to reconstruct past episodes of CH_4_ emission.

The sulphur isotope composition (δ^34^S) of biogenic carbonates was analysed previously to reconstruct the seawater sulphur isotope age curve (Burdett *et al*.^24^; Kampschulte *et al*.^25^). Further, the planktonic foraminiferal δ^34^S signature was used to study variations of the sulphur cycle in the early Cenozoic (Rennie *et al*.^26^). As confirmed by culturing experiments (Paris *et al*.^27^), the δ^34^S value of carbonate-associated sulphate is representative of the water in which the organisms calcify (Burdett *et al*.^24^), but small species-specific offsets exist (Rennie *et al*.^26^). The incorporation of sulphate in the calcium carbonate lattice is still unclear, but calcite precipitation experiments and analysis of natural carbonates suggested that sulphate substitutes for the carbonate ion (Staudt *et al*.^28^; Pingitore *et al*.^29^). In biogenic carbonates, sulphur is also contained in the shell (or skeleton) organic matrix (Lorens *et al*.^30^; Dauphin *et al*.^31^; Glock *et al*.^32^).

**Figure 1:**
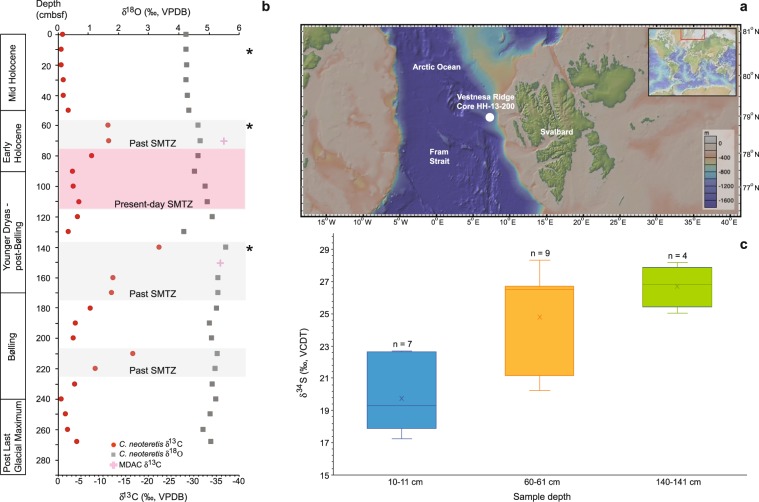
Sample location and *Cassidulina neoteretis* isotopic composition. (**a)** Map showing the location of Core HH-13-200. The map was generated using the software GeoMapApp, version 3.6.10. (http://www.geomapapp.org). (**b)** Carbon isotope ratios (δ^13^C) of CH_4_-derived authigenic carbonates and δ^13^C and δ^18^O values of *C. neoteretis* from core HH-13-200. Data are from Schneider *et al*.^13^. Present (red shading) and past (grey shading) positions of the sulphate-methane transition zone (SMTZ) are after Schneider *et al*.^13^. Asterisks indicate the sediment depth of the samples analysed for this study. (**c)** Box plot of the mean δ^34^S of *C. neoteretis* grouped by sampling depths. The median is represented by the central bar, whereas the mean is denoted with a ‘x’. Whiskers show minimum-maximum range of data. Cmbsf = cm below seafloor; VPDB = Vienna Pee Dee Belemnite; MDAC = methane-derived authigenic carbonates; VCDT = Vienna Canyon Diablo Troilite; n = number of specimens analysed.

In this study, we report *in situ* benthic foraminiferal δ^34^S data obtained by ion microprobe in order to test the hypothesis that the benthic foraminiferal δ^34^S can be used to infer the flux (diffusive vs. advective) of paleo CH_4_ emissions and the timing of CH_4_ seepage. Samples were collected in the Arctic Ocean, at a site of known gas hydrates and seepage of a mixture of microbial and thermogenic CH_4_ (Vestnesa Ridge; 78.981°N; 7.061°E; 1205 m water depth) (Panieri *et al*.^33^; Bünz *et al*.^34^) (Fig. 1a). We focused on samples for which past migrations of the SMTZ were well constrained (Fig. 1b). All the samples analysed belonged to the foraminiferal species *Cassidulina neoteretis*. We chose to focus on this species because of its relevance in paleoceanographic reconstructions (Consolaro *et al*.^12^; Schneider *et al*.^13^; Panieri *et al*.^22^) and its high abundance in the sampling region (Wollenburg and Mackensen^35^; Wollenburg and Mackensen^36^).

## Results

### *Cassidulina neoteretis* isotopic composition

Seven, nine, and four *C. neoteretis* were analysed from samples located at 10–11, 60–61, and 140–141 cmbsf (cm below seafloor), respectively. Each shell was analysed two or more times by ion microprobe, depending on the shell size, the exposed shell surface, and its position relative to the epoxy and the contaminant phases (i.e., pyrite, sediment) observed inside the chambers of many of the mounted specimens (Supplementary Figs. 1, 2; Supplementary Tables 1 and 2). Our data revealed a high δ^34^S intra-shell, and intra- and inter- sample variability (Supplementary Figs. 1, 2). Interestingly, the ion microprobe data collected also hinted at a variability in the cycle-by-cycle δ^34^S values in some of the spots analysed, in particular in shells from deeper sediment intervals (Supplementary Fig. 3). It is possible that this represents natural δ^34^S variations due to the analysis of different shell components (i.e., calcite, shell organic matrix, secondary overgrowth); however, the analytical uncertainty is too high to state this conclusively (Supplementary Fig. 3).

The δ^34^S signature of each shell was calculated by averaging the δ^34^S values of all the spots measured in the given shell. Single spot δ^34^S values ranged from ~13‰ to ~31‰, whereas single shell δ^34^S values ranged from ~17‰ to ~28‰ (Supplementary Fig. 2). The representative δ^34^S of each sample was calculated by averaging the δ^34^S values of all the shells measured in the given sample (Fig. 1c; Table 1). Sample δ^34^S values are distinct at a statistically significant level as confirmed by a Kruskal-Wallis test (H = 10.798, p < 0.005, df = 2). Further testing shows that only sample 10–11 cmbsf is statistically different from the other two samples (see Methods).

**Table 1:** List of samples used in this study.

Sample ID – Core HH-13-200	Sample depth (cmbsf)	# shells analysed for Mg/Ca (EDS)	# shells analysed for δ^34^S (SIMS)	δ^34^S_VCDT_	1 SD	δ^13^C_VPDB_	δ^18^O_VPDB_
Sect.1 10–11 cm	10–11	5	7 (27)	19.75	2.13	–0.49	4.21
Sect. 1 60–61 cm	60–61	5	9 (32)	24.79	3.06	–10.94	4.62
Sect. 2 40–41 cm	140–141	6	4 (8)	26.70	1.30	–22.18	5.52

Analyses were conducted on *Cassidulina neoteretis* shells. δ^34^S data are from this study; see Supplementary Tables 1 and 2 for the complete dataset. The numbers in parentheses represent the number of data points collected for each sample. Bulk foraminiferal δ^13^C and δ^18^O data are from Schneider *et al*.^13^. The analytical precision for these measurements was estimated to be better than ±0.08‰ and ±0.03‰ for oxygen and carbon, respectively (Schneider *et al*.^13^). Cmbsf = cm below seafloor; EDS = energy-dispersive x-ray spectroscopy; SIMS = secondary ion mass spectrometry; VCDT = Vienna Canyon Diablo Troilite; SD = standard deviation; VPDB = Vienna Pee Dee Belemnite.

**Figure 2:**
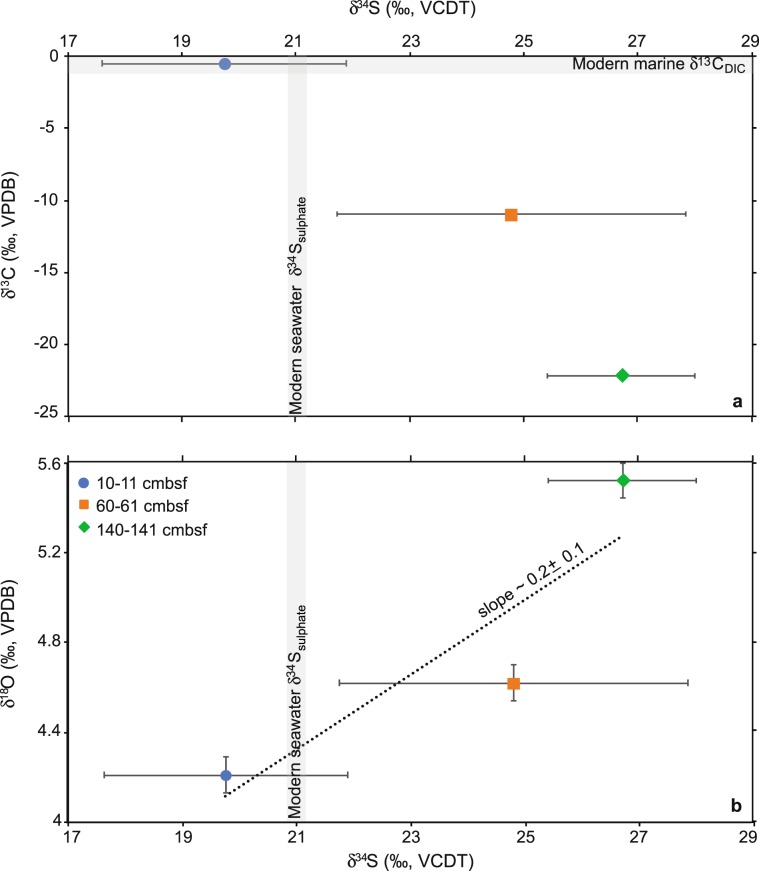
Stable isotope composition *of Cassidulina neoteretis*. (**a)** Plot of δ^13^C and δ^34^S and (**b)** plot of δ^18^O and δ^34^S values of *C. neoteretis* samples from different depths. Bulk δ^13^C and δ^18^O values are from Schneider *et al*.^13^ and are reported in Table 1. The mean δ^34^S value by sampling depth is calculated from the means of individual foraminifera. Error bars are as follows: analytical precision for δ^13^C and δ^18^O; 1 standard deviation for δ^34^S. The δ^13^C errors are smaller than the symbols. Modern marine DIC δ^13^C signature after Ravelo and Hillaire-Marcel^17^. Modern seawater sulphate δ^34^S value after Rees *et al*.^47^. Modern seawater δ^18^O is out of scale. VCDT = Vienna Canyon Diablo Troilite; VPDB = Vienna Pee Dee Belemnite; DIC = dissolved inorganic carbon; cmbsf = cm below seafloor.

The δ^34^S values of the samples analysed were compared to bulk δ^13^C and δ^18^O measured on *C. neoteretis* specimens from the same samples (Schneider *et al*.^13^). The δ^34^S and bulk δ^13^C values of *C. neoteretis* are negatively correlated (Fig. 2a). The correlation between δ^34^S and bulk δ^18^O is positive and consistent with previous studies (Antler *et al*.^37^; Feng *et al*.^38^). For our dataset, the slope of the δ^18^O-δ^34^S correlation is ~0.2 (Fig. 2b).

**Figure 3:**
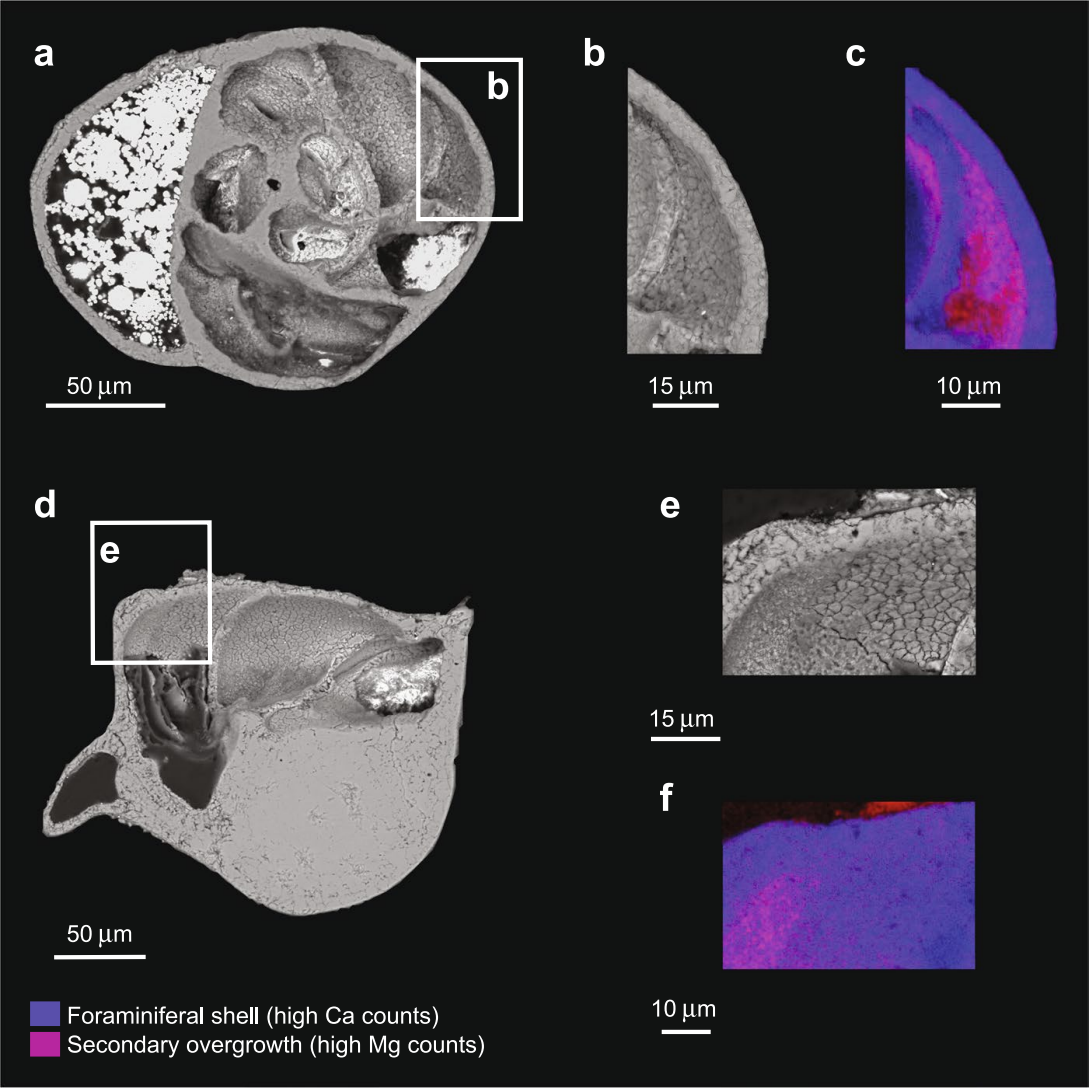
Examples of diagenetic alterations of *Cassidulina neoteretis* shells. (**a)** Backscatter electron (BSE) image of a specimen from sample 60–61 cm below seafloor (cmbsf). (**b**) BSE image and **(c)** X-ray map (Mg, red; Ca, blue) by energy dispersive x-ray spectroscopy (EDS) of the area outlined in **(a)**. (**d)** BSE image of a specimen from sample 140–141 cmbsf. Detail of the image shown in (**d**) as BSE image (**e**) and EDS map (**f**).

Microscopy and spectroscopy analysis showed the presence of sediment and pyrite inside some of the shells measured by ion microprobe; however, sulphur- and iron-rich particles were not observed neither in close proximity to, nor inside of, the ion microprobe pits. Electron microscopy and energy dispersive x-ray spectroscopy (EDS; i.e., magnesium and calcium content) analyses of additional *C. neoteretis* shells confirmed that secondary overgrowth was present on shells from 60–61 and 140–141 cmbsf (Fig. 3). The comparison of the lower δ^34^S values of foraminifera from 10–11 cmbsf and those of foraminifera from 60–61 and 140–141 cmbsf suggests that the presence of secondary overgrowth influenced the δ^34^S signatures of the shells located deeper in the sediment column. However, it was not possible to quantify the contribution of this secondary overgrowth on the sulphur isotopic composition of the specimens analysed.

## Discussion

The isotopic composition of pore water sulphate is much heavier at CH_4_ seep sites compared to non-seep sediments (e.g., δ^34^S values up to 70.8‰ vs. 20.7–23.4‰) (Aharon and Fu^39^). This enrichment is a consequence of sulphur isotope fractionation during sulphate reduction coupled to AOM. The analysis of *C. neoteretis* from different geochemical horizons (Fig. 1b) shows that *C. neoteretis* δ^13^C and δ^34^S values at 10–11 cmbsf agree with modern seawater DIC δ^13^C and sulphate δ^34^S (Fig. 2a), implying that no CH_4_ oxidation coupled to sulphate reduction was recorded by these foraminiferal shells. This finding is consistent with pore water profiles of the core analysed (Hong *et al*.^40^). On the contrary, the *C. neoteretis* of samples deeper in the sediment column (samples 60–61 and 140–141 cmbsf) are characterized by more negative δ^13^C and more positive δ^34^S values, mirroring the changes of the DIC δ^13^C and sulphate δ^34^S consequent to sulphate-driven CH_4_ degradation (Fig. 2a). Although our dataset revealed a negative correlation between the foraminiferal δ^13^C and δ^34^S, it was recently demonstrated that a positive correlation between δ^13^C and δ^34^S might be recorded in diagenetic carbonates (i.e., dolomite) as a consequence of high methanogenic activity in the geological past (Meister *et al*.^41^). More data are needed in order to explore if a similar signal can be recorded by diagenetically-altered foraminifera, as well.

Sulphate reduction rates influence the sulphate oxygen and sulphur isotopic compositions, which can be tracked by the slope in a sulphate δ^18^O vs. δ^34^S plot (Aharon and Fu^39^; Böttcher *et al*.^42^; Antler *et al*.^43^). At higher sulphate reduction rates, the δ^18^O of the residual sulphate pool increases more slowly compared to the pool δ^34^S resulting in a shallow slope; at lower rates, the opposite occurs, causing a steeper slope. As sulphate reduction proceeds, the shape of the slope can change as the sulphate δ^18^O approaches an asymptotic value. The difference in the evolution of the oxygen and sulphur isotope systems is connected to the isotope exchanges happening during microbial sulphate reduction (Aharon and Fu^39^; Antler *et al*.^43^). Interestingly, the sulphate δ^18^O-δ^34^S slope is different in environments characterized by CH_4_ advection (as opposed to diffusion) because of the influence of CH_4_ flux on the isotopic fractionation and exchange that happen during the microbial sulphate reduction steps (Antler *et al*.^37^). This unique signal can be preserved in fossil records, such as authigenic barite and carbonates (Antler *et al*.^37^; Feng *et al*.^38^).

In benthic foraminifera, the δ^18^O values are mainly influenced by the temperature, seawater δ^18^O value (δ^18^O_sw_), and vital effects, the last of which are negligible when a record is built analysing one species (Ravelo and Hillaire-Marcel^17^; Katz *et al*.^44^). To check whether the bulk *C. neoteretis* δ^18^O values of this study carry signals of temperature and/or δ^18^O_sw_ changes through time, we compared these data with a bulk *C. neoteretis* δ^18^O record from a gravity core collected on the western Svalbard margin, at a similar water depth as the core used in this study (Consolaro *et al*.^12^). The comparison between the bulk *C. neoteretis* δ^18^O values included in this study and coeval δ^18^O values from Consolaro *et al*.^12^ suggests that the δ^18^O values of our samples did not record changes in temperature and/or δ^18^O_sw_, but they were probably influenced by other processes (i.e., sulphate reduction coupled to AOM), as hypothesized here.

In foraminiferal shells, the presence of sulphur was reported in the calcite lattice (as sulphate) and organic matter within the shell (i.e., glycosaminoglycans and proteins) (Paris *et al*.^27^; Glock *et al*.^32^; Van Dijk *et al*.^45^; Geerken *et al*.^46^). Thus, the high variability in the *C. neoteretis* δ^34^S dataset (Fig. 1c; Supplementary Fig. 2) could be a result of the analysis of different phases, such as calcite, organic lining, and authigenic carbonates (secondary overgrowth), during data collection. Overall, we interpreted the δ^34^S signal measured in our samples as an enrichment in the ^34^S isotope due to sulphate reduction processes coupled to AOM in samples 60–61 and 140–141 cmbsf. Indeed, it would be beneficial to confidently distinguish the δ^34^S signature of the sulphate reduction process as recorded by the foraminifera from the signal contributed by the diagenetic overgrowth, since the occurrence of secondary overgrowth was confirmed by EDS analysis of samples 60–61 and 140–141 cmbsf (Fig. 3). Unfortunately, with the data available this is not possible (see Methods). However, one approach to assess the influence of the secondary overgrowth to the benthic foraminiferal pristine calcite δ^34^S would be to remove the data points having a δ^34^S signature equal to (or lower than) the modern seawater sulphate δ^34^S value (∼21‰; Rees *et al*.^47^) from the data collected on samples affected by sulphate-driven AOM (i.e., samples 60–61 and 140–141 cmbsf). We note that this method would affect only the sample 60–61 cmbsf, for which the δ^34^S signature would not increase significantly (i.e., ∼0.6‰; from 24.79‰ to 25.36‰). In addition, this approach would mask what we consider to be a natural intra- and inter-shell isotopic variability of seep foraminifera, in agreement with other studies (Rathburn *et al*.^14^; Sen Gupta and Aharon^18^; Panieri *et al*.^21^). Based on our data, we suggest that a δ^34^S signature between ∼25–27‰ as measured in diagenetically-altered benthic foraminifera is indicative of sulphate reduction coupled to AOM at the SMTZ.

In the following, we evaluate the possibility to use the slope of the benthic foraminiferal δ^18^O-δ^34^S correlation to infer the flux of past CH_4_ emission, which, to the best of our knowledge, has not been evaluated before. The slope of the δ^18^O-δ^34^S correlation as observed in our samples is ~0.2. Following Antler *et al*.^37^, we considered our slope a shallow slope implying that *C. neoteretis* recorded a sulphate δ^34^S growing faster than the δ^18^O. Based on the values of the sulphate δ^18^O-δ^34^S slope observed in environments characterized by CH_4_ advection compared to those with no or diffusive CH_4_ transport (0.28–0.66 vs. >0.58) (Antler *et al*.^37^), we interpret the slope of the benthic foraminiferal δ^18^O-δ^34^S correlation as a signal of CH_4_ advection at the sampling site. This is in agreement with the core sampling location (active pockmark with flares) and pore water profiles suggesting a strong CH_4_ flux at the site investigated (Hong *et al*.^40^).

The validity of the sulphate δ^18^O-δ^34^S slope to investigate microbial metabolism in sediments has recently been questioned (Antler and Pellerin^48^), but it can still provide important information regarding the biochemistry of sediments when used in a site-specific approach and in combination with other proxies (Antler and Pellerin^48^). For instance, our data demonstrate the possibility to reconstruct the migration of the SMTZ and infer the flux (i.e., diffusive vs. advective) of paleo CH_4_ emissions combining the benthic foraminiferal δ^13^C record with the slope in δ^18^O-δ^34^S. In geological records, the possibility to distinguishing between CH_4_ advection and diffusion (or degradation of organic matter) can provide new insight regarding the impact of marine CH_4_ to the carbon cycle (cf. Dickens^49^).

Dating CH_4_ seepage in fossil records is challenging. One approach is using the U/Th geochronology of authigenic carbonate crusts (Crémière *et al*.^8^). However, this method cannot always be applied because it requires pure calcite, a sufficient quantity of those elements, and abundant samples. Providing an age for SMTZ migrations, and associated processes as recorded by the isotopic composition of diagenetically altered foraminifera, is difficult because the precipitation of authigenic carbonates might not be correlated to the age of the host sediment (Schneider *et al*.^13^; Panieri *et al*.^22^). However, this could be resolved using the slope of the benthic foraminiferal δ^18^O-δ^34^S correlation (Fig. 2b). In environments characterized by CH_4_ advection, the slope of the benthic foraminiferal δ^18^O-δ^34^S correlation can be used as a novel approach to indirectly date fossil CH_4_ emissions because of the role of the CH_4_ flux in regulating the SMTZ depth (Borowski *et al*.^50^). At sites characterized by a high CH_4_ flux the SMTZ is rather shallow. In this case, the formation of a secondary overgrowth could be considered (almost) syn-sedimentary providing the opportunity to place the CH_4_ signal as recorded by benthic foraminifera in a stratigraphic context.

We test this hypothesis by looking at the stratigraphy of the samples analysed for this study, focusing on the samples 60–61 cmbsf and 140–141 cmbsf because the isotopic compositions of *C. neoteretis* show episodes of CH_4_ discharge at these horizons (Fig. 1b). Following our interpretation of the slope of the *C. neoteretis* δ^18^O-δ^34^S correlation (i.e., CH_4_ advection at the sampling site), CH_4_ emissions were coeval to the age of the hosted sediments, which according to the stratigraphic interpretation of the sedimentary record, were deposited during the early Holocene and Younger-Dryas-post Bølling (Schneider *et al*.^13^). Three lines of evidence support our inference. At 60–75 and 140–175 cmbsf, the presence authigenic carbonate nodules and high Ca/Ti and Sr/Ti suggest precipitation of aragonite, one of the phases typical of methane-derived authigenic carbonates that precipitate within a SMTZ close to the seafloor (Schneider *et al*.^13^; Aloisi *et al*.^51^). Also, precipitation of aragonite is an indication of strong CH_4_ flows and oxidation in sediments (Luff *et al*.^52^), independently supporting our interpretation of the slope of the *C. neoteretis* δ^18^O-δ^34^S correlation, indicating that CH_4_ seepage occurred (roughly) at the same time of sediment deposition.

The isotopic signature of the *C. neoteretis* analysed might have been influenced by migrations of the SMTZ subsequent to sediment deposition. More specifically, it is possible that the foraminiferal isotopic composition was influenced by an additional layer of secondary overgrowth that precipitated on the shells after burial, in addition to the secondary overgrowth that formed (almost) at the time of sediment deposition. In this scenario, the samples 60–61 and 140–141 cmbsf were located within the SMTZ multiple times during different time intervals. This scenario is certainly possible, but unlikely because of the connection and interplay among factors regulating the depth and thickness of the SMTZ, like sulphate concentration in the water column, sulphate penetration into the sediment, CH_4_ production, vicinity to gas hydrates, rate of carbon burial, sediment porosity and permeability, and the presence and morphology of gas migration conduits (Knittel and Boetius^6^; Boetius and Wenzhöfer^53^; Plaza-Faverola and Keiding^54^; Liu *et al*.^55^).

In summary, foraminifera are excellent carriers of information because they are geographically widespread, inhabit CH_4_ seeps, are preserved in fossil records, and can be easily retrieved through coring (Panieri *et al*.^11^; Consolaro *et al*.^12^; Schneider *et al*.^13^; Bernhard *et al*.^56^). In addition, in CH_4_-rich environments, the foraminiferal shells constitute a template for secondary overgrowth, which provide clues regarding past CH_4_ seepage (Schneider *et al*.^13^; Panieri *et al*.^21^). Our data show a negative correlation between the *C. neoteretis* δ^13^C and δ^34^S values that we interpret to be a consequence of changes in seawater DIC δ^13^C and sulphate δ^34^S due to sulphate-driven CH_4_ degradation. Furthermore, based on the slope of the δ^18^O-δ^34^S correlation as observed in our samples, we propose that CH_4_ advection occurred during the early Holocene and Younger-Dryas-post Bølling at our sampling site. This study represents the first application of the benthic foraminiferal δ^34^S to assess the flux and timing of paleo CH_4_ emissions. More data from sediments characterized by CH_4_ diffusion and advection, and high and low organic matter contents will be needed to further investigate the reliability of this proxy. However, based on the results obtained, this approach holds great promises to investigate CH_4_ dynamics in fossil records and their interactions with the marine carbon cycle.

## Methods

### Materials and sample preparation

Fossil specimens of the benthic foraminiferal species *Cassidulina neoteretis* (Seidenkrantz^57^) were selected from three sediment samples along one gravity core (HH-13-200; Table 1). The sample selection was based on the available pore water and bulk δ^13^C and δ^18^O foraminiferal stable isotope data (Schneider *et al*.^13^) (Fig. 1b), which were correlated with the *in situ* δ^34^S data obtained in this study. The core was collected at Vestnesa Ridge in 2013. The core age model was based on well-established stratigraphic tie-points for the western Svalbard continental margin (Schneider *et al*.^13^). Detailed information about core sampling and sample processing can be found in Schneider *et al*.^13^.

Foraminiferal specimens were handpicked under a stereomicroscope from the sediment fraction >100 μm. Twenty-five *C. neoteretis* shells were mounted in epoxy (EpoxyCure®, Buehler) for ion microprobe analysis. The shells, together with the standard, were positioned at the centre of the mount. The mount was let dry overnight at room temperature and then it was gently polished by hand using a 1200 grit silicon carbide paper (Buehler), so to expose the interior of the foraminiferal shells. The mount was subsequently impregnated with epoxy to fill any exposed void and polished with 1 μm Al_2_O_3_ powder (Mark V Laboratories). We note that our protocol is similar to other published methodologies to prepare foraminiferal cross-sections for ion microprobe analysis (Glock *et al*.^58^; Kozdon *et al*.^59^), even if our sample preparation was optimized for mounting and polishing relatively small *C. neoteretis* specimens.

Prior to ion microprobe work, the mount was gold coated and each foraminiferal shell was imaged using a Zeiss Auriga scanning electron microscope (SEM) at the University of Rochester (Rochester, NY) using a voltage of 20 kV. The SEM gold coating was then removed using a polishing pad with no Al_2_O_3_ powder and the mount was sonicated for one second, carefully rinsed with deionized water, soaked in methanol for a few minutes, and let dry in a vacuum oven overnight. A new gold coating of the appropriate thickness was applied before isotope measurements.

Twenty *C. neoteretis* shells were selected for energy dispersive x-ray spectroscopy (EDS) analysis of magnesium and calcium. These shells were mounted in epoxy (EpoxyCure®, Buehler) and let dry overnight at room temperature. The epoxy mount was first gently cleaned by hand using a 600 grit silicon carbide powder and then manually polished using 6 μm, 3 μm, 1 μm, 0.25 μm, and 0.1 μm diamond suspension (Struers; DP-suspension P and DP-Lubricant Blue). The mount was then soaked in ethanol for a few minutes, dried, and carbon coated prior analysis. After preliminary SEM and EDS analyses, the mount was polished again, so to better expose the inside of the shell chambers. For this second polishing, the mount was cleaned by hand using a 600 grit silicon carbide powder and then automatically polished using 6 μm, 3 μm, and 1 μm diamond suspension (Struers; DP-suspension P and DP-Lubricant Blue). Following the polishing, the mount was soaked in ethanol for a few minutes, dried, and carbon coated prior analysis.

### Standard preparation

For ion microprobe analysis, we used a chip of a natural coral as the standard. Although the coral mineralogy differs from that of foraminifera (aragonite vs. calcite), this was the best standard at our disposal as no commercially available standards exist for ion microprobe analysis of sulphur isotopes in calcite. The coral used was collected in 2010 on a beach on the north shore of Oahu Hawaii (USA), where it was washed up. After collection, several small pieces were broken from the coral piece for combustion analysis. Each fragment was crushed using a rotary mechanical crusher, grounded using a ceramic mortar and pestle, and centrifuged at 1500 rpms for 30 minutes at 25 °C. After that, the supernatant fluid was decanted, the remaining powder was dried at 60 °C, and then was soaked in 15% H_2_O_2_ at room temperature for 1–2 weeks. During this time, each sample was periodically ultrasonicated at 80 °C. Measurements of the coral sulphur isotope composition via combustion were performed at the Environmental Isotope Laboratory, Geosciences Department, University of Arizona (Tucson, AZ). The analysis of five samples yielded a coral δ^34^S _VCDT_ = 22.5‰ ± 0.43‰ (1 standard deviation [SD]; VCDT = Vienna Canyon Diablo Troilite). The analytical precision was ±0.15‰ (1 SD) based on the analysis of barium sulphate standard (QG).

The coral chip used during ion microprobe work was first treated with H_2_O_2_ and then hand polished on a 1200 grit silicon carbide paper prior mounting. Once ready, the coral fragment was mounted together with the foraminiferal samples and prepared as described in the previous section.

### Foraminiferal sulphur isotope analysis by ion microprobe

Sulphur isotope analyses were conducted at the University of California Los Angeles (UCLA; Los Angeles, CA), using the CAMECA *ims* 1290 ion microprobe. Compared to other instruments (e.g., isotope ratio mass spectrometer), an ion microprobe allows to collect data at high spatial resolution. This enabled us 1) to avoid pyrite inclusions and sediment infill, which were present in several of the shells available for analysis (Supplementary Fig. 1), and 2) to minimize the amount of shell needed for analysis, because bulk δ^34^S measurements would have required a lot of species-specific shells that were not available for the samples investigated.

We measured twenty shells in two, two-day long analytical sessions (September 2018 and January 2019). After the first analytical session, the mount was examined with a SEM at the University of Rochester, cleaned with acetone, and soaked in ethanol before being gold coated again prior to the second analytical session. Single-spot sulphur isotope analyses were conducted using a ~0.07 nA Cs^+^ primary beam. Data were acquired in multicollection mode, with two electron multipliers (axial EM and H2). Mass resolution was set at 4,000 to isolate the peaks of interest from interferences. Prior to signal collection, each spot was pre-sputtered for 90 seconds to achieve sputtering equilibrium. Each spot analysis consisted of 50 cycles for the coral standard and 200 cycles for the foraminiferal shells. Each cycle included a counting time of 5 seconds for sulphur isotope. For the coral standard, the average ^32^S counts rate on the EM detector was ~122,000 cps (counts per second), whereas the ^34^S count rate on the H2 detector was ~5,500 cps. The stability of the instrument was constantly monitored and the instrumental mass fractionation was determined by analysing the standard several times throughout each analytical session. The internal precision was better than 0.8‰ (1 standard error [SE]). External reproducibility on the standard was ~2‰ or better (1 SD).

### Ion microprobe data reduction

Isotope ratios of the unknowns were calibrated using a coral standard with a known sulphur isotopic composition on the VCDT scale (22.5 ± 0.43‰, Vienna Canyon Diablo Troilite; determined by combustion analysis); conventional delta notation was used and values were calculated relative to the VCDT standard (${R}_{{\rm{VCDT}}}$ = 0.0441638) (Ding *et al*.^60^):

$${\delta }^{34}{\rm{S}}=\left(\frac{{{\rm{R}}}_{{\rm{Sample}}}^{{\rm{Measured}}}}{{\rm{\alpha }}\,{{\rm{R}}}_{{\rm{VCDT}}}}-1\right)\times 1000$$

with

$${\rm{R}}=\frac{{}^{34}{\rm{S}}}{{}^{32}{\rm{S}}}$$

$$\alpha =\frac{{{\rm{R}}}_{{\rm{Standard}}}^{{\rm{Measured}}}\,}{{{\rm{R}}}_{{\rm{Standard}}}^{{\rm{True}}}\,}.$$

Instrumental drift was monitored by frequent analyses of the coral standard throughout each session. During session 1 (September 2018) and the first day of session 2 (January, 7, 2019), no instrumental drift was observed; therefore, isotope ratios were corrected using one α obtained for a given day. For the second day of session 2 (January, 8, 2019), a standard-unknown-standard bracketing approach was applied. Ion microprobe data are reported in Supplementary Tables 1 and 2.

### Foraminiferal microscopy and spectroscopy analyses

The observation of the foraminiferal shells analysed by ion microprobe was conducted by SEM and EDS to verify the location of each spot and the presence of possible authigenic carbonates, pyrite, and carbon-rich regions in the area analysed. Scanning electron microscopy images and EDS maps were collected using a SEM Zeiss Merlin VP Compact equipped with an EDS x-max 80 system by Oxford instruments, combined with the analytical software AZtec at The Arctic University of Norway (Tromsø, Norway). Compositional data on each shell was acquired for 2–3 hours, depending on the sample, using a voltage of 20 kV.

As previously shown (Panieri *et al*.^21^), it can be challenging to identify the presence of authigenic carbonates (secondary overgrowth) precipitated on the foraminiferal shell through microscopy, because the secondary overgrowth can be morphologically indistinguishable from the calcite precipitated by the foraminifera. However, authigenic carbonates can be differentiated from the primary foraminiferal calcite based on their higher magnesium content (Torres *et al*.^16^; Panieri *et al*.^21^; Schneider *et al*.^61^). Unfortunately, the gold coating on the mount analysed by ion microprobe prevented us from reliably assessing the magnesium contents of the foraminifera measured. We decided not to make a second polish of the mount in order to best preserve the foraminiferal surface analysed for further studies. Because of this, we prepared a second mount containing twenty *Cassidulina neoteretis* shells picked from the same sediment samples used for the δ^34^S measurements. The analysis of this mount was conducted at The Arctic University of Norway using a Hitachi Tabletop Microscope TM-3030 equipped with a Bruker Quantax 70 EDS Detector combined with the analytical software Quantax 70. Compositional data were acquired on sixteen specimens, for 10–30 minutes/shell using a voltage of 15 kV.

### Statistical analyses

The linear regression for the δ^18^O-δ^34^S plot was calculated using the least square method.

The δ^34^S difference among samples was evaluated using the non-parametric Kruskal-Wallis test. Although this test does not assume that the data are normally distributed, it requires the observations be independent and the data have the same distribution. We tested for this last condition using the Levene’s test, which showed that the variances were not significantly different among samples (p > 0.05).

The post-hoc Dunn’s test was used for pairwise comparisons. The results indicate that the sample 10–11 cmbsf was statistically different from the samples 60–61 and 140–141 cmbsf (p < 0.02 and p < 0.003, respectively) and that the sample 60–61 cmbsf was not significantly different from the 140–141 cmbsf one (p > 0.3). No corrections were made to the p-values.

## Supplementary information

Supplementary Information.

Datasets 1 and 2.

## Data Availability

Ion microprobe data are provided in Supplementary Tables 1 and 2. Additional SEM images and EDS maps are available from the corresponding author (C.B.) on reasonable request.
