# Supplementary material for: The benthic foraminiferal δ34S records flux and timing of paleo methane emissions
Source: Sci Rep. 2020 Jan 28;10:1304. doi: 10.1038/s41598-020-58353-4 (PMC6987089; doi:10.1038/s41598-020-58353-4)
Supplement: Supplementary file 1 — Supplementary Information. [file 41598_2020_58353_MOESM1_ESM.pdf]

Supplementary Information

The benthic foraminiferal $\delta^{34}S$ records flux and timing of paleo methane emissions

C. Borrelli[1,2,*], R. I. Gabitov[3], M.-C. Liu[4], A. T. Hertwig[4], and G. Panieri[2]

[1]*Department of Earth and Environmental Sciences, University of Rochester, Rochester, NY, USA*

[2] *CAGE - Centre for Arctic Gas Hydrate, Environment and Climate, Department of Geosciences, UiT The Arctic University of Norway, Tromsø, Norway*

[3] *Department of Geosciences, Mississippi State University, Mississippi State, MS, USA*

[4] *Department of Earth, Planetary, and Space Sciences, University of California Los Angeles, Los Angeles, CA, USA*

*corresponding author: cborrelli@ur.rochester.edu

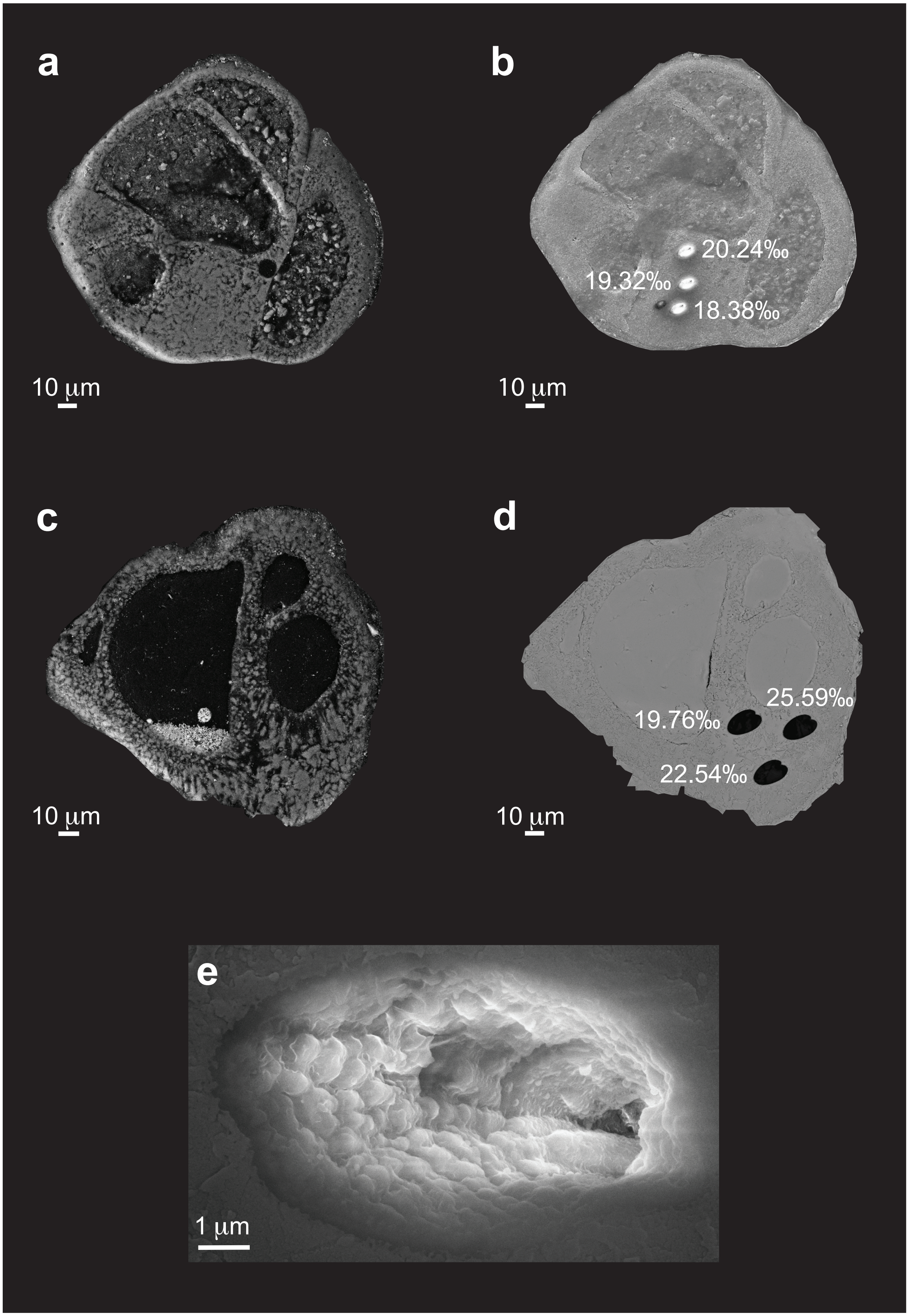

a
10 µm
b
20.24‰
19.32‰
18.38‰
10 µm
c
10 µm
d
25.59‰
19.76‰
22.54‰
10 µm
e
1 µm

Supplementary Figure 1. Examples of *Cassidulina neoteretis* shells prior and after ion microprobe analysis. a, c, Secondary electron images of *C. neoteretis* shells prior ion microprobe analysis. Samples are as follows: specimens 6 (**a**) and 10 (**c**) from sample 10-11 cm below seafloor. Note the presence of sediment inside the chambers of the shell in (**a**) and the presence of pyrite at the bottom of the left chamber of the shell in (**c**). **b,** Secondary electron image of the shell in (**a**) after analysis. **d,** Backscatter electron image of the shell in (**c**) after analysis. In (**b**) and (**d**), the $\delta^{34}S$ value of each ion microprobe spot is specified. **e,** Secondary electron image of an ion microprobe spot on a *C. neoteretis* shell.

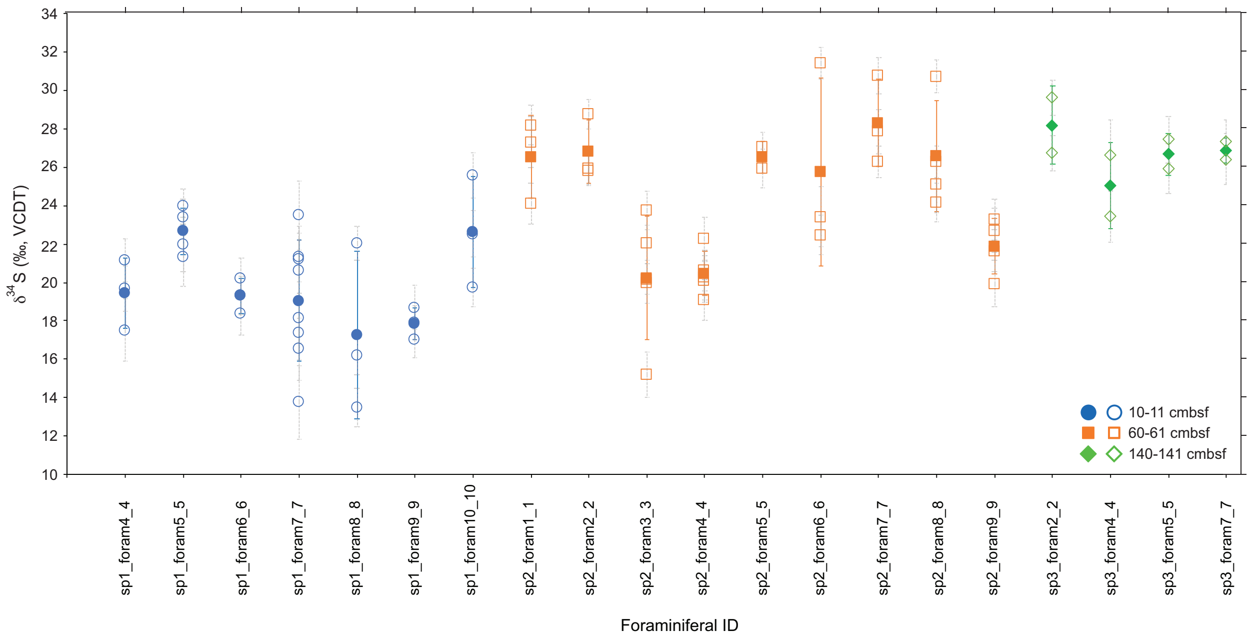

Supplementary Figure 2. Sulphur isotopic composition of single foraminiferal shells. All the samples analyzed belong to *Cassidulina neoteretis*. For each specimen, the mean $\delta^{34}S$ value (filled symbol) was calculated by averaging the $\delta^{34}S$ values of all the spots (blank symbols) measured in the given shell. Error bars are as follows: specimen mean $\delta^{34}S$ = 1 standard deviation (solid lines); single spot $\delta^{34}S$ value = 1 standard error (grey, dashed lines). The foraminiferal ID matches the first part of the analysis ID as specified in Supplementary Tables 1 and 2, where the ion microprobe data are reported. VCDT = Vienna Canyon Diablo Troilite; cmbsf = cm below seafloor.

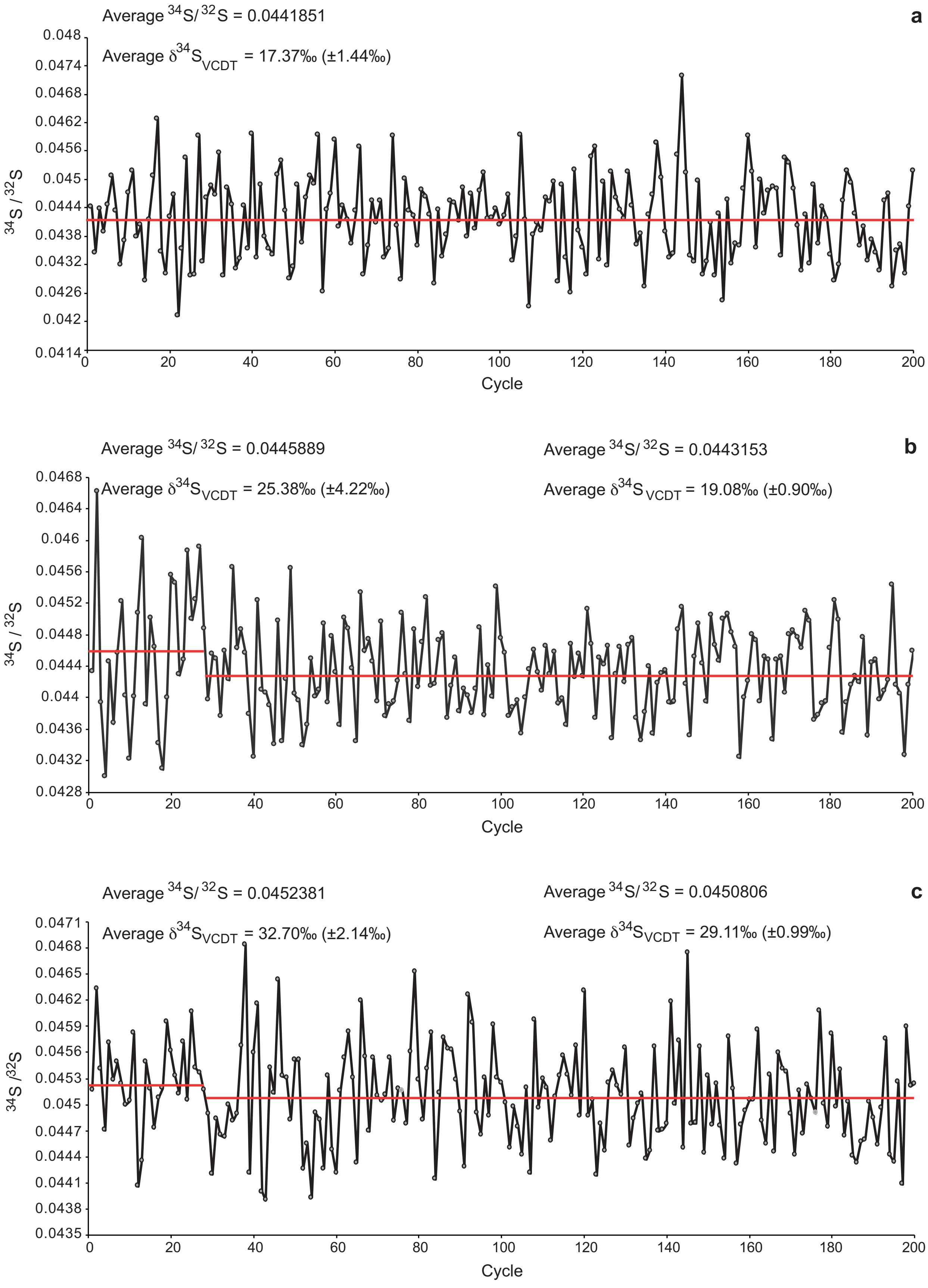
a
Average $^{34}S/^{32}S$ = 0.0441851
Average $\delta^{34}S_{VCDT}$ = 17.37‰ (±1.44‰)
$^{34}S/^{32}S$
Cycle
b
Average $^{34}S/^{32}S$ = 0.0445889
Average $\delta^{34}S_{VCDT}$ = 25.38‰ (±4.22‰)
Average $^{34}S/^{32}S$ = 0.0443153
Average $\delta^{34}S_{VCDT}$ = 19.08‰ (±0.90‰)
$^{34}S/^{32}S$
Cycle
c
Average $^{34}S/^{32}S$ = 0.0452381
Average $\delta^{34}S_{VCDT}$ = 32.70‰ (±2.14‰)
Average $^{34}S/^{32}S$ = 0.0450806
Average $\delta^{34}S_{VCDT}$ = 29.11‰ (±0.99‰)
$^{34}S/^{32}S$
Cycle

Supplementary Figure 3. $^{34}S/^{32}S$ signal vs. cycle number. a, Profile acquired from specimen 7, sample 10-11 cm below seafloor (cmbsf), spot 6. **b,** Profile acquired from specimen 9, sample 60-61 cmbsf, spot 3. **c,** Profile acquired from specimen 2, sample 140-141 cmbsf, spot 1. The errors among parentheses are the standard error of the mean. VCDT = Vienna Canyon Diablo Troilite.
